# A comparative study of extraction techniques for maximum recovery of glutamate decarboxylase (GAD) from *Aspergillus oryzae* NSK

**DOI:** 10.1186/1756-0500-6-526

**Published:** 2013-12-10

**Authors:** Audrey Lee Ying Yeng, Mohd Safuan Ab Kadir, Hasanah Mohd Ghazali, Raja Noor Zaliha Raja Abd Rahman, Nazamid Saari

**Affiliations:** 1 Faculty Biotechnology and Biomolecular Sciences, Universiti Putra Malaysia, 43400, UPM Serdang, Selangor, Malaysia; 2 Faculty of Food Science and Technology, University Putra Malaysia, 43400, Serdang, Selangor, D.E., Malaysia

**Keywords:** *Aspergillus oryzae*, Glutamate decarboxylase, γ-Amino-butyric acid, Sonication, Mechanical disruption, Enzymatic lysis, Chemical permeabilization

## Abstract

**Background:**

γ-Amino butyric acid (GABA) is a major inhibitory neurotransmitter of the mammalian central nervous system that plays a vital role in regulating vital neurological functions. The enzyme responsible for producing GABA is glutamate decarboxylase (GAD), an intracellular enzyme that both food and pharmaceutical industries are currently using as the major catalyst in trial biotransformation process of GABA. We have successfully isolated a novel strain of *Aspergillus oryzae* NSK that possesses a relatively high GABA biosynthesizing capability compared to other reported GABA-producing fungal strains, indicating the presence of an active GAD. This finding has prompted us to explore an effective method to recover maximum amount of GAD for further studies on the GAD’s biochemical and kinetic properties. The extraction techniques examined were enzymatic lysis, chemical permeabilization, and mechanical disruption. Under the GAD activity assay used, one unit of GAD activity is expressed as 1 μmol of GABA produced per min per ml enzyme extract (U/ml) while the specific activity was expressed as U/mg protein.

**Results:**

Mechanical disruption by sonication, which yielded 1.99 U/mg of GAD, was by far the most effective cell disintegration method compared with the other extraction procedures examined. In contrast, the second most effective method, freeze grinding followed by 10% v/v toluene permeabilization at 25°C for 120 min, yielded only 1.17 U/mg of GAD, which is 170% lower than the sonication method. Optimized enzymatic lysis with 3 mg/ml Yatalase^®^ at 60°C for 30 min was the least effective. It yielded only 0.70 U/mg of GAD. Extraction using sonication was further optimized using a one-variable-at-a-time approach (OVAT). Results obtained show that the yield of GAD increased 176% from 1.99 U/mg to 3.50 U/mg.

**Conclusion:**

Of the techniques used to extract GAD from *A. oryzae* NSK, sonication was found to be the best. Under optimized conditions, about 176% of GAD was recovered compared to recovery under non optimized conditions. The high production level of GAD in this strain offers an opportunity to conduct further studies on GABA production at a larger scale.

## Background

Glutamate decarboxylase (GAD) is a unique enzyme that catalyzes the irreversible conversion of L-glutamic acid (Glu) to γ-aminobutyric acid (GABA) and carbon dioxide through a single step α-decarboxylation pathway. The cofactor that is involved in the pathway is pyridoxal phosphate [1]. GABA, a major inhibitory neurotransmitter, participates in all functions of the central nervous system (CNS) by blocking nerve impulses, without which or at low level will cause neurological disorders such as Parkinson’s disease, Huntington’s chorea, cognitive impairment, and etc [1,2]. GABA also has additional physiological roles, which include lowering of blood pressure and cholesterol level, calming and tranquilizing influences, antidiabetic and diuretic effects, etc. [3-5]. Because of these therapeutic properties, GABA has the potential to be incorporated into commercially significant foods and pharmaceutical products. Since GAD is the specific enzyme that catalyzes the production of GABA, it could be an important addition to enzymes that are already used in the food and pharmaceutical industries, and thus the extraction of GAD from safe and reliable sources will play a crucial aspect in the production of GABA.

GAD can be found naturally in a rich diversity of organisms, ranging from microscopic organisms to higher plants and animals. It is primarily isolated from microbial sources including bacteria, yeasts and molds [6-9]. Among these sources, *Aspergillus oryzae* was frequently chosen for its ability to secrete various desired enzymes that are widely used in the production of fermented foods such as soysauce, vinegar etc [7]. GAD is reported as one of the enzymes that can be produced by this fungus [7,9]. A further recognition as a Generally Recognized as Safe (GRAS) microorganism by the United States Food and Drug Administration (FDA) and World Health Organization makes it an attractive GABA-producing candidate to be safely utilized in funcitonal food production on an industrial scale (WHO) [10,11]

In order to recover high amounts of GAD from the mycelia of *A. oryzae*, the method used to disintegrate the cells ideally should not affect the activity and properties of the enzyme [12]. The nature of GAD as an intracellular enzyme implies that an additional lysis step is required for the complete destruction of the cell wall in order to release the enzyme [13,14]. Various cell disruption methods, either mechanical or non-mechanical, have been employed to release the protein content of fungal cells [15-18]. Isolation of enzymes using non-mechanical means involves either chemical treatment or the use of lytic enzymes. The former affects the cells directly but may cause autolysis by intracellular enzymes, as has been reported for yeasts [19], while the latter is regarded as gentle to fungi cells with high specificity but they are costly in large-scale intracellular protein recovery [12,15]. Furthermore, the complex composition of fungal cell walls may require a treatment that consists of a mixture of different enzymes, thus it might not be efficient.

Mechanical disruption means disruption of cells without the addition of either chemicals or enzymes. Generally, this technique of cell breakage is economical and suitable for large-scale preparations. However, it produces excessive heat, which needs to be monitored and controlled [20]. Among various mechanical methods, sonication is the most commonly applied mechanical methods for its ease of up-scaling process and it requires neither sophisticated instrument nor extensive technical training [21]. Fungal cells suspended in a liquid buffer are broken apart by intense sonic pressure waves generated by an ultrasonicator. The pressure waves create micro bubbles that grow and coalesce, vibrate violently and eventually causing implosions that generate high energy shock wave sufficient to disrupt cells [22]. Homogenization is another appropriate technique that may work well with cells that are difficult to be lysed e.g. fungi cells [20]. The cells are disrupted by high shear forces exerted from external pressure actions e.g. grinding via bead mill, high pressure homogenizer, pestle and tube homogenizer, etc [22,23].

There are very few studies performed on the recovery of GAD from *A. oryzae* and thus the search for an effective technique that recovers high amounts of GAD from the fungus remains a significant area for exploratory study. In the present study, the efficiency of different cell disruption methods in maximizing the recovery of intracellular GAD from *A. oryzae* NSK was investigated.

## Methods

### Fungal strain

*Aspergillus oryzae* NSK (GenBank:JN381021) with high GABA biosynthesizing property was isolated from a koji sample produced by a local soy sauce processing plant and stored at -40°C on a PDA slant containing 20 (w/v) glycerol [24].

### Materials

γ-Amino butyric acid (GABA), 5′-pyridoxal phosphate (5′PLP) and calcium chloride dihydrate were obtained from Sigma Aldrich Inc. (St. Louis, MO, USA). Methanol, toluene, hexane, acetone, chloroform, potato dextrose agar (PDA), Tween 80, glucose monohydrate, L-glutamic acid, magnesium sulfate heptahydrate, sodium carbonate, monopotassium phosphate, and Lactophenol Cotton Blue were purchased from Merck (Germany). Bacto™ yeast extract was from Becton, Dickinson & Company (USA). Yatalase^®^ enzyme was purchased from Takara Shuzo Co. (Otsu, Shiga, Japan). Triton X-100, 3-[(3-Cholamidopropyl)dimethylammonio]-1-propanesulfonate (CHAPS), sodium dodecyl sulfate (SDS), and citric acid monohydrate were purchased from Acros Organics (Geel, Belgium). Trisodium citrate was sourced from Fisher Scientific (United States). All chemicals were of analytical reagent grade.

### Conidia preparation and submerged cultivation of *A. oryzae* NSK

Conidia of *A. oryzae* NSK were prepared from a 5-day old culture on PDA (incubated at room temperature) by scraping them into 5.0 mL of 5 (w/v) Tween 80 under sterile condition. The number of conidia was determined using a hemocytometer. Conidia at 10^4^ mL^-1^ cell concentration was inoculated in a medium (pH 5.5) containing 5 (w/v) glucose, 0.4% (w/v) L-glutamic acid, 0.1% (w/v) MgSO_4_7H_2_O, 0.15% (w/v) KH_2_PO_4_, 0.6% (w/v) yeast extract and 0.2% (w/v) CaCl_2_.2H_2_O. The medium was then incubated for 3 days in an orbital shaker at 200 rpm and 37°C. Fungal mycelia that germinated from the conidia were harvested by filtration through a Whatman No. 4 nylon membrane filter and the filtrate was collected for the analysis of possible extracellular GAD activity [24].

### Extraction of glutamate decarboxylase (GAD) from fungal mycelia

Mycelia at 10% (w/v) (wet weight basis) suspension were used throughout GAD extraction procedures. The mycelia were suspended in 50 mM citrate buffer (pH 5.5) and extraction was performed according to the procedures as described in Sections 2.5-2.9. GAD activity and soluble protein concentration were measured during the screening stage in order to monitor the efficiency of different extraction methods. Comparison of efficiency is made based on the specific activity. The best method was selected for further optimization. All experiments were carried out in triplicate.

### Enzymatic lysis

Enzymatic lysis of mycelia was performed using different concentrations (3, 5, 10, 15, 20, & 25 mg/mL) of Yatalase^®^, an enzyme preparation that consists mainly of chitinase, chitobiase and β-1,3 glucanase, dissolved in 50 mM sodium citrate buffer, pH 5.5. Cell lysis was performed based on four parameters: cell concentration (5, 10, 20, 30, 40, 50, & 60% w/v), incubation temperature (25, 30, 35, 40, 45, 50, 55, 60, & 70°C), and incubation time (15, 30, 60, 120, 180, 240, & 300 min). Each of the parameter was optimized by maintaining all the other parameters at a constant value i.e. 10% (w/v) cell concentration, 60 min and 30°C incubation time and temperature, and 10 mg/mL of Yatalase^®^. At the end of the experiment, the cell suspension was centrifuged at 14,000 × *g* for 30 min at 4°C and the supernatant was analyzed for GAD activity (Takara Bio Inc.).

### Chemical permeabilization

The effect of organic solvents on cell permeabilization was performed according to Klimek-Ochab et al [15] with some modification. Five types of organic solvents namely ethanol, toluene, acetone, chloroform and butanol were tested. A 10% (w/v) suspension of pre-ground mycelia according to the Section 2.7, was prepared in 1 mL of 50 mM citrate buffer, pH 5.5, containing 10% v/v organic solvent and shaken at room temperature (25°C) with different incubation time (30, 60, & 120 min). The cell suspension was then centrifuged at 14,000 × *g* for 30 min at 4°C and the supernatant was analyzed for GAD activity.

For detergent permeabilization, pre-ground mycelia were suspended separately in 0.2% (v/v) aqueous solutions of detergents (Triton X-100, CHAPS, Tween 80, and SDS) to give a working concentration of 10% cell (w/v), and incubated with shaking (200 rpm) at room temperature (25°C) under three different incubation time (30, 60, & 120 min) [15].

### Cell breakage with liquid nitrogen

Mycelia were frozen rapidly using liquid nitrogen and ground into fine powder using a pre-chilled mortar and pestle. The ground mycelia were suspended in 50 mM citrate buffer, pH 5.5, to give a working concentration of 10% (w/v) and vortexed for 1 min. The cell suspension was then centrifuged at 14,000 × *g* for 30 min at 4°C and supernatant was analyzed for GAD activity.

### High speed homogenization

Mycelia (10% w/v) were suspended in 50 mL of cold citrate buffer (4°C). The suspension was placed in a salt ice-water bath and the cells were disrupted using a high speed homogenizer (Miccra D-9, Germany) at different speed (14,000, 16,000, 18,000, 21,000, & 24,000 rpm). The cell suspension was then centrifuged at 14,000 × *g* for 30 min at 4°C and the supernatant was analyzed for enzyme activity.

### Sonication

Mycelia (10% w/v) were suspended in 10 mL of cold citrate buffer (4°C) and disrupted with an ultrasonic homogenizer (Biologics, Inc., USA) at 50% of pulser, 30 W for a total of 10 min in a salt ice-water bath. Sonication was stopped every 30 s to allow the cell suspension to cool down for 30 s. Centrifugation was performed at 14,000 × *g* for 30 min and the resulting supernatant was examined for GAD activity.

### One-Variable-at-a-Time (OVAT) methodology for optimizing the sonication protocol

Sonication was performed in an ice-water bath using a Biologics Inc. (USA) ultrasonic homogenizer at 20 kHz. All sonication parameters, except the one being optimized, were maintained at 50% pulser, 10% cell concentration, 10 mL cell suspension in 50 mM citrate buffer, pH 5.5, and 30 W acoustic power. The investigated variables were cell concentration [2, 4, 6, 8, 10, 12, and 14% (w/v)], cell suspension volume (4, 6, 8, 10, and 12 mL), acoustic power (10, 20, 30, 40, and 50 W) and sonication time (5, 10, 15, 20, and 25 min) on a discontinuous mode (30 s of sonication cycle followed by a cooling interval of 30 s). The distance between the sonicator tip and the base of sample holder was maintained at a distance of 2 cm throughout the experiment. All experiments were carried out in triplicate.

### Determination of GAD activity

Enzyme solution (0.1 mL) was mixed with 0.9 mL of reaction mixture comprising 50 mM L-glutamic acid and 50 μM PLP in 100 mM citrate buffer at pH 5.5 and incubated at 37°C for 30 min. The reaction was terminated by the addition of 1.0 mL of 0.5 M Na_2_CO_3_ solution [10]. The mixture was then centrifuged at 14,000 × *g* for 30 min at 4°C. The concentration of the product of reaction (GABA) was determined according to the method of Rossetti and Lombard (1996) [25]. Results of assay were obtained in triplicate. One unit of GAD activity is defined as the amount of GAD that produces 1 μmol of GABA per min per mL extract (U/mL) while the specific activity is defined as GAD activity (U) per mg protein (U/mg).

### Determination of GABA

The concentration of the product of reaction (GABA) was determined according to the method of Rossetti and Lombard (1996) [25]. A 100 μL aliquot of supernatant (or of standard solution of GABA) was dried under vacuum. The residue was dissolved in 20 μL of ethanol-water-triethylamine mixture (2:2:1 v/v) and evaporated to dryness under vacuum. A 30 μL volume of ethanol-water-triethylamine-PITC (7:1:1:1 v/v/v) mixture was then added to the residue and allowed to react for 20 min at room temperature to form PITC-GABA. The excess reagent was then removed under vacuum. The dry residue containing PITC-GABA was dissolved in 200 μL of the mobile phase, consisting of a mixture of 60% Solution A (aqueous solution of 8.205 g sodium acetate, 0.5 mL triethylamine, and 0.7 mL acetic acid in 1000 mL) adjusted to pH 5.8, 28% Solution B (deionized water), and 12% Solution C (acetonitrile). Gradient HPLC separations were performed on a Shimadzu LC 20AT apparatus, consisting of pump system, a CT0-10ASVP model oven with a 20 μL injection loop injector, and a Model SPD-M20A PDA detector, in conjunction with a DELL Model DELL Optiplex integrator. A Hypersil Gold C-18 column (250×4.6 mm I.D., particle size 5 μm; Thermoscientific, Meadow, UK) was used for separation purposes. Separation was performed at flow rate of 0.6 mL/min, 37°C and wavelength used was UV 254 nm.

### Protein assay

Protein concentration was determined according to Bradford [26] with a protein assay kit (Merck, USA) and bovine serum albumin was used as the protein standard.

### Microscopic examination of fungal mycelia

Mycelia before and after all mechanical disruptions were examined using a light microscope after staining with Lactophenol Cotton Blue [27]. Magnification was between 4- to 40-fold.

### Statistical Analysis

All experiments were conducted in triplicate. Statistical analysis of the data was carried out using Statistical Analysis System (SAS, Institute Inc, 1988).

## Results and discussion

### Comparison of enzymatic lysis, chemical permeabilization and mechanical disruption on GAD yields

Recently, a novel strain of *A. oryzae* NSK with high GABA biosynthesizing property was isolated from koji samples provided by an industrial soy sauce processing plant [24]. In order to determine the capability of this strain in the bioconversion of L-glutamate to GABA, it is necessary to develop an efficient method that is able to recover high amounts of GAD. In this study, three different extraction techniques namely enzymatic lysis, chemical permeabilization, and mechanical disruption were examined.

#### Enzymatic lysis

A commercial lytic enzyme, Yatalase^®^, capable of releasing GAD from a fungal source has been suggested elsewhere [7]. Studies were conducted to assess the efficiency of enzymatic lysis by evaluating the effect of incubation time (min), Yatalase^®^ concentration (mg/ml), cell concentration (% w/v), and incubation temperature (˚C) on the release of GAD from fungal cell (Additional file 1). Subsequently, the results demonstrated that maximum GAD yield was achieved when 30% (w/v) of cell concentration was incubated with 3 mg/mL of Yatalase at 60°C for 30 min and the final GAD yield obtained was 0.70 U/mg (Table 1). A plausible explanation to high yields of GAD at temperature of 60°C may be due to thermal-induced autolysis of fungus cells rather than enzyme-induced release [28], since Yatalase^®^’s optimal temperature is 37°C (Clontech Laboratories, Inc.). These results also revealed an interesting finding of which the GAD extracted from *A. oryzae* NSK may possess excellent thermo stability although this is in contrast to the results obtained by Tsuchiya et al [10] where GAD purified from *A. oryzae* was found to be thermally stable below 40°C.

**Table 1:** Comparison of different cell disintegration techniques in the recovery of GAD

	**Total activity (U)**	**Total protein (mg)**	**Specific activity (U/mg)**
**Enzymatic lysis**			
Yatalase^®^ (3 mg/ml, 30% w/v cell concentration, 30 min, 60˚C)	0.4 ± 0.01	0.61 ± 0.02	0.70 ± 0.02
**Chemical permeabilization**			
*Solvent permeabilization*			
Toluene, 10% (v/v), 120 min, 25°C	0.09 ± 0.02	0.14 ± 0.03	0.97 ± 0.05
Chloroform, 10% (v/v), 120 min, 25°C	0.15 ± 0.04	0.17 ± 0.07	0.88 ± 0.06
Butanol, 10% (v/v), 60 min, 25°C	0.06 ± 0.02	0.08 ± 0.04	0.78 ± 0.02
Ethanol, 10% (v/v), 120 min, 25°C	0.08 ± 0.01	0.19 ± 0.05	0.42 ± 0.04
Acetone, 10% (v/v), 60 min, 25°C	0.04 ± 0.03	0.17 ± 0.04	0.24 ± 0.07
*Detergent permeabilization*			
Triton X-100, 0.2% (v/v), 120 min, 25°C	0.13 ± 0.04	0.14 ± 0.07	0.93 ± 0.08
CHAPSO, 0.2% (v/v), 120 min, 25°C	0.10 ± 0.07	0.17 ± 0.02	0.62 ± 0.04
Tween 80, 0.2% (v/v), 120 min, 25°C	0.09 ± 0.02	0.26 ± 0.05	0.36 ± 0.05
SDS, 0.2% (v/v), 120 min, 25°C	0.04 ± 0.02	0.15 ± 0.01	0.28 ± 0.08
**Mechanical disruption**			
Sonication (10% w/v cell concentration, 50% pulser, 30 W, 10 min)	6.7 ± 0.02	3.40 ± 0.04	1.99 ± 0.38
High speed Homogenization (10% w/v cell concentration, 18,000 rpm, 30 min)	3.52 ± 0.02	14.0 ± 0.01	0.25 ± 0.03

Values are shown as the means ± SD over three independent replications.

#### Chemical permeabilization

The efficiency of solvent permeabilization was examined using five different solvents with hydrophobicity (Log P) ranging from -0.24 to 2.69 (Table 1). Different types of detergents were also assessed for their efficiency as cell permeabilizing agents in releasing GAD from pre-ground mycelia. According to Taubert et al [12], the main location at which cell permeabilization occurs is the outer layer of the hyphae that serves as the diffusion barrier against permeabilizing agents. Based on this concept, the fungal mycelia were first disrupted by grinding the mycelia frozen by liquid nitrogen prior to incubation with the permeabilizing agents. Among all of the solvents used in cell permeabilization, 10% toluene-assisted extraction (Log P = 2.14) at 25°C for 120 min yielded the highest GAD (0.97 U/mg), followed by chloroform (Log P = 2.69) with a specific activity of 0.88 U/mg. The use of acetone (Log P = -0.24) led to a yield of 75.25% lesser than toluene-assisted extraction.

In detergent permeabilization, the highest GAD yield of 0.93 U/mg was obtained when the fungus was treated with 0.2% Triton X-100 at 25°C for 120 min (Table 1). On the other hand, SDS yielded the least amount of intracellular enzyme, which might be due to the possibility of destructive impacts that were contributed by the ionic interactions between the enzymes and detergent residues [15].

#### Mechanical disruption

High speed homogenization and sonication were employed as the mechanical means of cell disruption in this study. High speed homogenization recorded an optimum GAD yield of 0.25 U/mg under homogenization speed of 18,000 rpm. On the other hand, disruption of 10% (w/v) cell concentration by sonication at 50% of pulser and 30 W of acoustic power for 10 min has produced GAD yield of 1.99 U/mg. The results of all of the cell disruption methods are summarized in Table 1 and it shows that sonication method was the most efficient method to recover intracellular GAD from *A. oryzae*. The GAD yield of sonication method was 284% higher compared to other cell disruption methods namely enzymatic lysis and about 221% higher compared to treatment with detergent and solvent.

**Figure 1:**
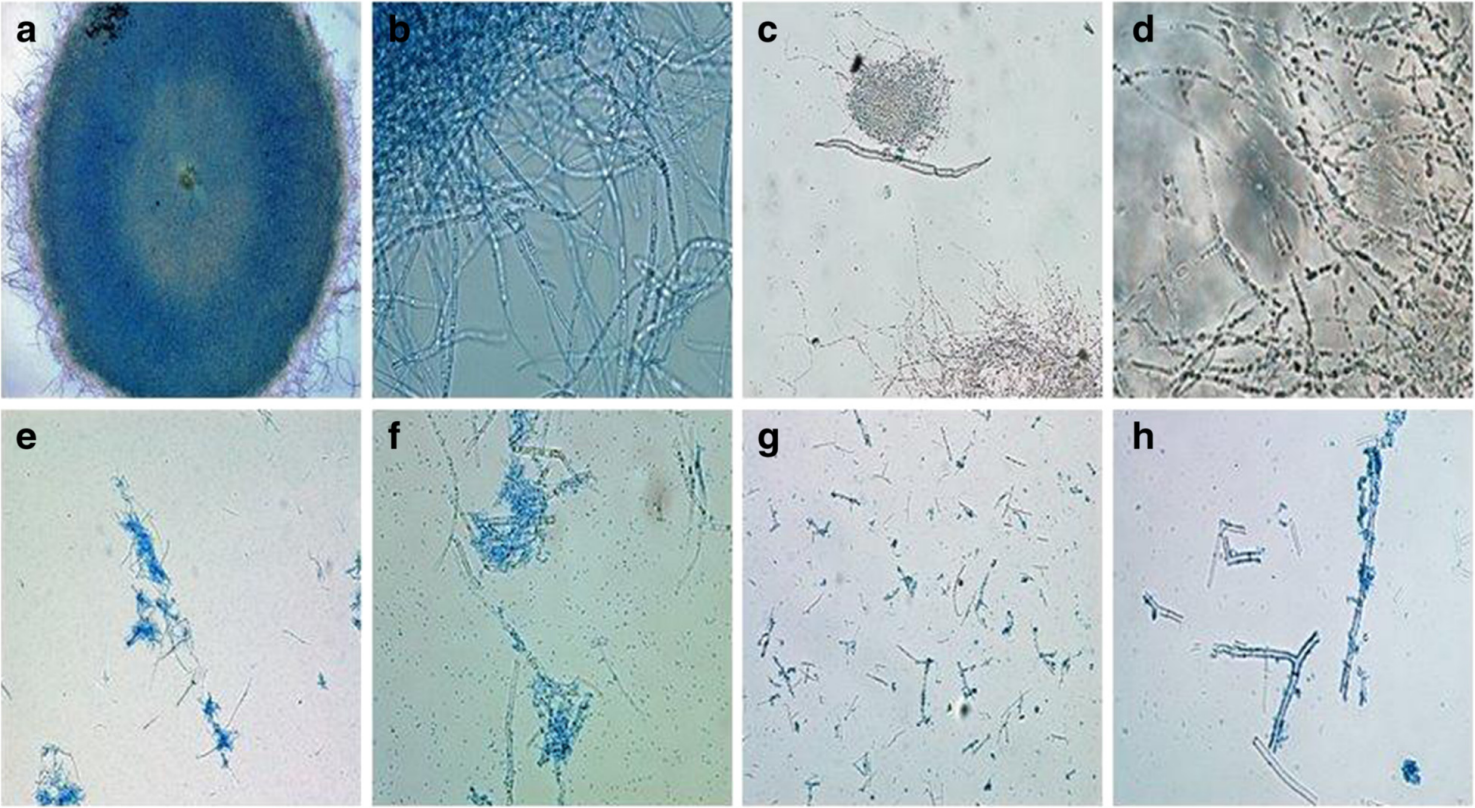
**Light microscopy of mycelia from *A. oryzae *NSK before and after cell disruption. (a)** Original structure of mycelia before cell disruption with X4 magnification; **(b)** Original structure of mycelia before cell disruption with X40 magnification; **(c)** Enzymatic lysis by Yatalase^®^ (30 min, 3 mg/ml, 30% w/v cell concentration, 60˚C) with X10 magnification; **(d)** Enzymatic lysis by Yatalase^®^ with X40 magnification; **(e)** Cell permeabilized by toluene (0.2% (v/v) toluene, cell concentration 10% (w/v), 120 min, 25°C) with X10 magnification; **(f)** Cell permeabilized by toluene with X40 magnification; **(g)** After sonication with X10 magnification (cell concentration 12% (w/v), 8 mL suspension volume, 50 W & 20 min) and **(h)** After sonication with X40 magnification.

Furthermore, the degree of cell disruption by various cell disruption methods was also determined by microscopic examination of cell debris. Prior to cell disruption, *A. oryzae* appears as filamentous mycelia pellets under 10X magnification (figure 1a) and its filaments appears as interwoven complex of hyphae under 40X magnification (figure 1b). Among all the cell disruption techniques, the mycelia under sonication were found to be completely disintegrated and uniformly dispersed in the suspension (Figure 1g). On the other hand, the formation of clustered hyphae resulted from toluene permeabilization shows partial disruption evidently (1e & 1f). As for enzymatic lysis (Figure 1c and 1d), the hyphae were seen largely intact under microscopy, incidentally, it also produced the lowest yield of GAD among all the methods employed. From these observations, it is noteworthy that complete cell disruption gave higher yield as it allows more intracellular content to be released and in turn increasing the GAD yield. The efficiency of sonication method in GAD extraction have led to further optimization of its critical parameters in order to recover the maximum amount of GAD from fungal cells.

### Optimization of sonication protocols

**Figure 2:**
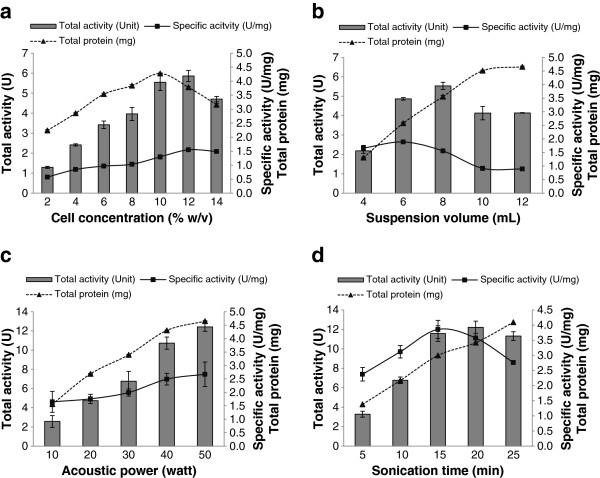
**Optimization of sonication protocols on the recovery of GAD. (a)** Effect of cell concentration (% w/v) on GAD release by suspending the mycelia in 10 mL of citrate buffer at pH 5.5 and sonicated at 20 Khz, 50 W for 10 min; **(b)** Effect of buffer suspension volume (mL) on GAD release by sonicate 10% (w/v) of mycelia at 20 Khz, 50 W for 10 min; **(c)** Effect of acoustic power (watt) on GAD release by suspending mycelia (10% w/v) in 10 mL of citrate buffer at pH 5.5 and sonicated at 20 Khz for 10 min; **(d)** Effect of sonication time (min) on GAD release by suspending mycelia (10% w/v) in 10 mL of citrate buffer at pH 5.5 and sonicated at 20 Khz and 50 W. Results represent the mean (± SD) of three experiments.

Four critical parameters of sonication including cell concentration (% w/v), buffer suspension volume (mL), acoustic power (watt), and sonication time (min) were optimized for the maximum recovery of GAD. The effect of cell concentration (% w/v) ranging from 2% to 14% was determined. As shown in Figure 2a, the maximum yield of GAD (1.55 U/mg) was obtained when 12% of cell concentration was applied but the yield decreased significantly (*P < 0.05*) when cell concentration was further increased. Similar observation was also reported by Saptarshi and Lele [17] whom demonstrated that the recovery of intracellular L-asparaginase is directly proportional to the cell concentration but reduction of enzyme yield occurred beyond the optimum cell concentration. This phenomenon could be due to the distortion of frequency on sonic wave’s passages and severe attenuation on sound intensity under highly saturated condition [16,29]. Both effects cause the reduction in the cavitation zone and consequently the disruption of mycelia cell becomes less effective, which can be observed from Figure 2a where GAD activity and soluble proteins were started to decline at 12% of cell concentration.

The effect of suspension volume is shown in Figure 2b, where the highest GAD activity was obtained at 6 mL of suspension volume. Increasing the volume further significantly decreased the recovered activity. A similar trend was reported by other researchers during the recovery of intracellular proteins such as L-asparaginase and hepatitis B core antigen [17,18,30]. The reason for this phenomenon is that at lower suspension volume, more sonic energy was dissipated per unit of volume causing the generation of extremely strong cavitation that led to the alteration of protein molecules and hence enzyme inactivation [31]. In contrast, reduction of GAD yield at bigger suspension volume might be due to the dilution of energy dissipated per unit of volume that reduced the cavitation and thus leading to the formation of large eddies [32]. Eddies with scales larger than a cell simply carry the cell from place to place and are not intense enough to cause effective cell disruption [17,18,32].

The effect of acoustic power (Watt) ranging from 10–50 W was also evaluated. Figure 2c depicts a linear relationship between the released GAD and acoustic power. Similar observation was also reported in the work of Ho et al. [18] where the intracellular hepatitis B-core antigen was released constantly without thermal denaturation when acoustic power was increased up to 180 W under proper control of temperature [18].

In addition to cell concentration and cell suspension volume, sonication time ranging from 5 min to 25 min on the release of GAD was also assessed. As shown in Figure 2d, the enzyme activity increased concomitantly with sonication time and the optimum yield (3.86 U/mg) was obtained following sonication for 15 min. Prolonged sonication significantly (*p < 0.05*) reduced the recovery of GAD. Several studies suggested that free radicals generated from the pyrolysis of water and ionization during prolonged sonication could inactivate the recovered intracellular enzymes [21,33].

In comparison to other findings, *A. oryzae* NSK may require a longer sonication time (15 min) for complete disruption of cells based on the microscopic evidence (Figure 1) in order to release the intracellular GAD. Cell disruption process is largely dependent on the physical strength of microorganism cell wall and the location of intracellular enzyme [34]. According to Bowman and Free [35], the cell wall of *Aspergillus* sp. contain between 10 and 20% of chitin that enhances its tensile strength and cell wall rigidity, which implies that a longer sonication time is required to disrupt the integrity of the fungal cell wall. By combining the four optimum crucial parameters for sonication, the yield of GAD was successfully enhanced by 176% from 1.99 U/mg to 3.50 U/mg. Therefore, it can be concluded that sonication is the best approach to extract high yields of GAD from the fungal strain compared to other reported approaches such as enzymatic solubilization, non-ionic detergent, mechanical lysis and bead mill [7,8]. The capability of *A. oryzae* NSK for producing high amount of GAD is useful for upscaling GABA production.

## Conclusion

Ultrasonication was found to be the best approach to release the intracellular GAD from *Aspergillus oryzae* NSK compared to chemical and non-chemical cell disruption methods. The optimized sonication protocols employed (6 mL buffer suspension volume, 12% (w/v) cell concentration and 50 W acoustic power for 15 minutes) has successfully increased the yield of GAD by 176% from an unoptimized conditions. This is the first report pertaining to the comparison experimentation and optimization of GAD extraction procedures from a GABA-producing *A. oryzae*.

## Abbreviations

GAD: Glutamate decarboxylase; GABA: γ-aminobutyric acid; CHAPS: 3-[(3-Cholamidopropyl)dimethylammonio]-1-propanesulfonate; SDS: Sodium dodecyl sulfate; PITC: Phenylisothiocyanate.

## Competing interests

The authors declare that they have no competing interests.

## Authors’ contributions

ALYY performed all data acquisition, data analyses, and manuscript writing. MSBA-K isolated the fungal strain of *Aspergillus oryzae* NSK and optimized its fermentation media. HMG, RNZRAR and NS contributed to conception of the study, experimental design, and revision of the manuscript. All authors read and approved the final manuscript.

## Authors’ information

ALYY is master student and MSAK is currently a PhD student. HMG and NS are professors of enzyme technology and food biochemistry respectively at Faculty of Food Science and Technology while RNZRAR is a professor at the Faculty of Biotechnology and Biomolecular Sciences.

## Supplementary Material

Additional file 1 **Optimization of the condition of enzymatic lysis on the recovery of GAD by using Yatalase^®^.** (a) Effect of incubation time (min) of Yatalase^®^ (10 mg/ml) on 10% (w/v) of mycelia at 30°C in 50 mM Citrate buffer, pH 5.5. (b) Effect of concentration of lytic enzyme on mycelia (10% w/v) at 30°C for 60 min. (c) Effect of cell concentration (% w/v) on the enzymatic lysis of Yatalase^®^ (10 mg/ml) at 30°C for 60 min. (d) Effect of incubation temperature on the enzymatic lysis of Yatalase^®^ (10 mg/ml) at 10% (w/v) of mycelia for 60 min. Results represent the mean (± SD) of three experiments. Click here for file

## References

[B1] Krnjević K Chemical nature of synaptic transmission in vertebrates Physiol Rev 1974 6 418 540

[B2] Wong GT Bottiglieri T Snead C GABA, γ-hydroxybutyric acid, and neurological disease Ann Neurol 2003 6 3 12 10.1002/ana.10696 12891648

[B3] Brandão ML De Aguiar JC Graeff FG GABA mediation of the anti-aversive action of minor tranquilizers Pharmacol Biochem Behav 1982 6 Suppl 3 397 402 6123116 10.1016/0091-3057(82)90441-5

[B4] Shimada M Hasegawa T Nishimura C Kan H Kanno T Nakamura T Matsubayashi T Anti-hypertensive effect of gamma-aminobutyric acid (GABA)-rich Chlorella on high-normal blood pressure and borderline hypertension in placebo-controlled double blind study Clin Exp Hypertens 2009 6 Suppl 4 342 354 19811362 10.1080/10641960902977908

[B5] Soltani N Qiu H Aleksic M Glinka Y Zhao F Liu R Li Y Zhang N Chakrabarti R Ng T Jin T Zhang H Lu WY Feng ZP Prud'homme GJ Wang Q GABA exerts protective and regenerative effects on islet beta cells and reverses diabetes Proc Natl Acad Sci U S A 2011 6 Suppl 28 11692 11697 21709230 10.1073/pnas.1102715108 PMC3136292

[B6] Ueno Y Hayakawa K Takahashi S Oda K Purification and characterization of glutamate decarboxylase from *Lactobacillus brevis* IFO 12005 Biosci Biotech Biochem 1997 6 Suppl 7 1168 1171 10.1271/bbb.61.1168 9255981

[B7] Tsuchiya K Nishimura K Iwahara M Purification and characterization of glutamate decarboxylase from *Aspergillus oryzae* Food Sci & Tech Res 2003 6 Suppl 3 283 287

[B8] Yang SY Lin Q Lu ZX Lu FX Bie XM Zou XK Sun LJ Characterization of a novel glutamate decarboxylase from *Streptococcus salivarius* ssp. *thermophilus* Y2 J Chem Tech & Biotech 2008 6 855 861 10.1002/jctb.1880

[B9] Kato Y Kato Y Furukawa K Hara S Cloning and Nucleotide Sequence of the Glutamate Decarboxylase-encoding Gene gadA from *Aspergillus oryzae* Biosci Biotech Biochem 2002 6 Suppl 12 2600 2605 10.1271/bbb.66.2600 12596854

[B10] Partial List of Enzyme Preparations That are Used in Foods, US Food and Drugs Administration http://www.fda.gov/food/ingredientspackaginglabeling/gras/enzymepreparations/ucm084292.htm

[B11] FAO/WHO Evaluation of certain food additives and contaminants Thirtieth Report of the Joint FAO/WHO Expert Committee on Food Additives 1987 15 16 http://whqlibdoc.who.int/trs/WHO_TRS_751.pdf 3111105

[B12] Taubert J Krings U Berger RG A comparative study on the disintegration of filamentous fungi J Microbiol Meth 2000 6 225 232 10.1016/S0167-7012(00)00194-9 11044566

[B13] Strigácová J Chovanec P Liptaj T Hudecová D Turský T Simkovic M Varecka L Glutamate decarboxylase activity in *Trichoderma viride* conidia and developing mycelia Arch Microbiol 2001 6 32 40 10.1007/s002030000235 11271418

[B14] Kumar S Punekar N The metabolism of 4-aminobutyrate (GABA) in fungi Mycol Res 1997 6 Suppl 4 403 409

[B15] Klimek-Ochab M Brzezinska-Rodak M Zymanczyk-Duda E Lejczak B Kafarski P Comparative study of fungal cell disruption-scope and limitations of the methods Folia Microbiol 2011 6 469 475 10.1007/s12223-011-0069-2 21901292 PMC3189342

[B16] Belur PD Mugeraya G Nainegali B Release of cell associated tannase of *Serratia ficaria* DTC by sonication, surfactants and solvents Asian J Biotech 2011 6 91 97

[B17] Saptarshi SD Lele SS Application of evolutionary optimization technique in maximizing the recovery of L-asparaginase from *E. caratovovora* MTCC 1428 Glob J Biotech Biochem 2010 6 Suppl 2 97 105

[B18] Ho CW Chewa TK Ling TC Kamaruddin S Tan WS Tey BT Efficient mechanical cell disruption of *Escherichia coli* by an ultrasonicator and recovery of intracellular hepatitis B core antigen Proc Biochem 2006 6 1829 1834 10.1016/j.procbio.2006.03.043

[B19] Breddam K Beenfeldt T Acceleration of yeast autolysis by chemical methods for production of intracellular enzymes Appl Microbiol Biotechnol 1991 6 323 329 10.1007/BF00172720 22622933

[B20] Ahmed H Vasta GR Extraction of Protein Principles and Reactions of Protein Extraction, Purification, and Characterization 2005 Baltimore: CRC Press 17 21

[B21] Chisti Y Young MM Disruption of microbial cells for intracellular products Enz Microbiol Tech 1986 6 196 204

[B22] Middelberg APJ Process-scale disruption of microorganisms Biotech Adv 1995 6 Suppl 3 491 551 10.1016/0734-9750(95)02007-p 14536098

[B23] Ho CW Tan WS Yap WB Ling TC Tey BT Comparative evaluation of different cell disruption methods for the release of recombinant hepatitis B core antigen from *Escherishia coli* Biotechnol Bioproc Eng 2008 6 Suppl 5 577 583

[B24] Ab Kadir MS Enhancement of γ-aminobutyric acid from Aspergillus oryzae in batch fermentation 2011 Master thesis: Universiti Putra Malaysia, Food Science Department

[B25] Rosetti V Lombard A Determination of glutamate decarboxylase by high performance liquid chromatography J chroma 1996 6 63 67 10.1016/0378-4347(96)88202-8 8798913

[B26] Bradford MM A rapid and sensitive method for the quantitation of microgram quantities of protein utilizing the principles of protein-dye binding Anal Biochem 1976 6 248 254 10.1016/0003-2697(76)90527-3 942051

[B27] Kormanik PP McGraw AC Schenck NC Quantification of vesicular-arbuscular mycorrhizae in plant roots Methods and Principles of Mycorrhizal Research 1982 Minnesota: APS press 37 40

[B28] Huang B Lin W Cheung PC Wu J Differential proteomic analysis of temperature-induced autolysis in mycelium of Pleurotus tuber-regium Curr Microbiol 2011 6 Suppl 4 1160 1167 21161227 10.1007/s00284-010-9838-4

[B29] Gogate PR Wilhelm AM Pandit AB Some aspects of the design of sonochemical reactors Ultrason Sonochem 2003 6 325 330 10.1016/S1350-4177(03)00103-2 12927607

[B30] Feliu JX Villa VA Cubarsi R Optimized release of recombinant protein by ultrasonication of *Escherichia coli* Biotechnol Bioeng 1998 6 Suppl 5 36 40 10.1002/(sici)1097-0290(19980605)58:5<536::aid-bit10>3.0.co;2-9 10099290

[B31] Dagbagli S Goksungur Y Optimization of β-galactosidase production using *Kluyveromyces lactis* NRRL Y-8279 by response surface methodology Electron J Biotechnol 2008 6 1 12

[B32] Doulah MS Mechanism of disintegration of biological cells in ultrasonic cavitation Biotechnol Bioeng 1977 6 Suppl 5 649 660 857952 10.1002/bit.260190504

[B33] Rachinskaya ZV Karasyova EI Metelitza DI Inactivation of glucose-6-phosphate dehydrogenase in solution by low and high frequency ultrasound Appl Biochem Microbiol 2004 6 120 128

[B34] Kuboi R Umakoshi H Takagi N Komasawa I Optimal disruption methods for the selective recovery of β-Galactosidase from *Escherichia coli* J Ferment Bioeng 1995 6 Suppl 4 335 341

[B35] Bowman SM Free SJ The structure and synthesis of fungal cell wall BioEssays 2006 6 Suppl 8 799 808 16927300 10.1002/bies.20441

