# Supplementary material for: A comparative study of extraction techniques for maximum recovery of glutamate decarboxylase (GAD) from Aspergillus oryzae NSK
Source: BMC Res Notes. 2013 Dec 10;6:526. doi: 10.1186/1756-0500-6-526 (PMC4029468; doi:10.1186/1756-0500-6-526)
Supplement: Additional file 1 — Optimization of the condition of enzymatic lysis on the recovery of GAD by using Yatalase®. (a) Effect of incubation time (min) of Yatalase® (10 mg/ml) on 10% (w/v) of mycelia at 30°C in 50 mM Citrate buffer, pH 5.5. (b) Effect of concentration of lytic enzyme on mycelia (10% w/v) at 30°C for 60 min. (c) Effect of cell concentration (% w/v) on the enzymatic lysis of Yatalase® (10 mg/ml) at 30°C for 60 min. (d) Effect of incubation temperature on the enzymatic lysis of Yatalase® (10 mg/ml) at 10% (w/v) of mycelia for 60 min. Results represent the mean (± SD) of three experiments. [file 1756-0500-6-526-S1.pdf]

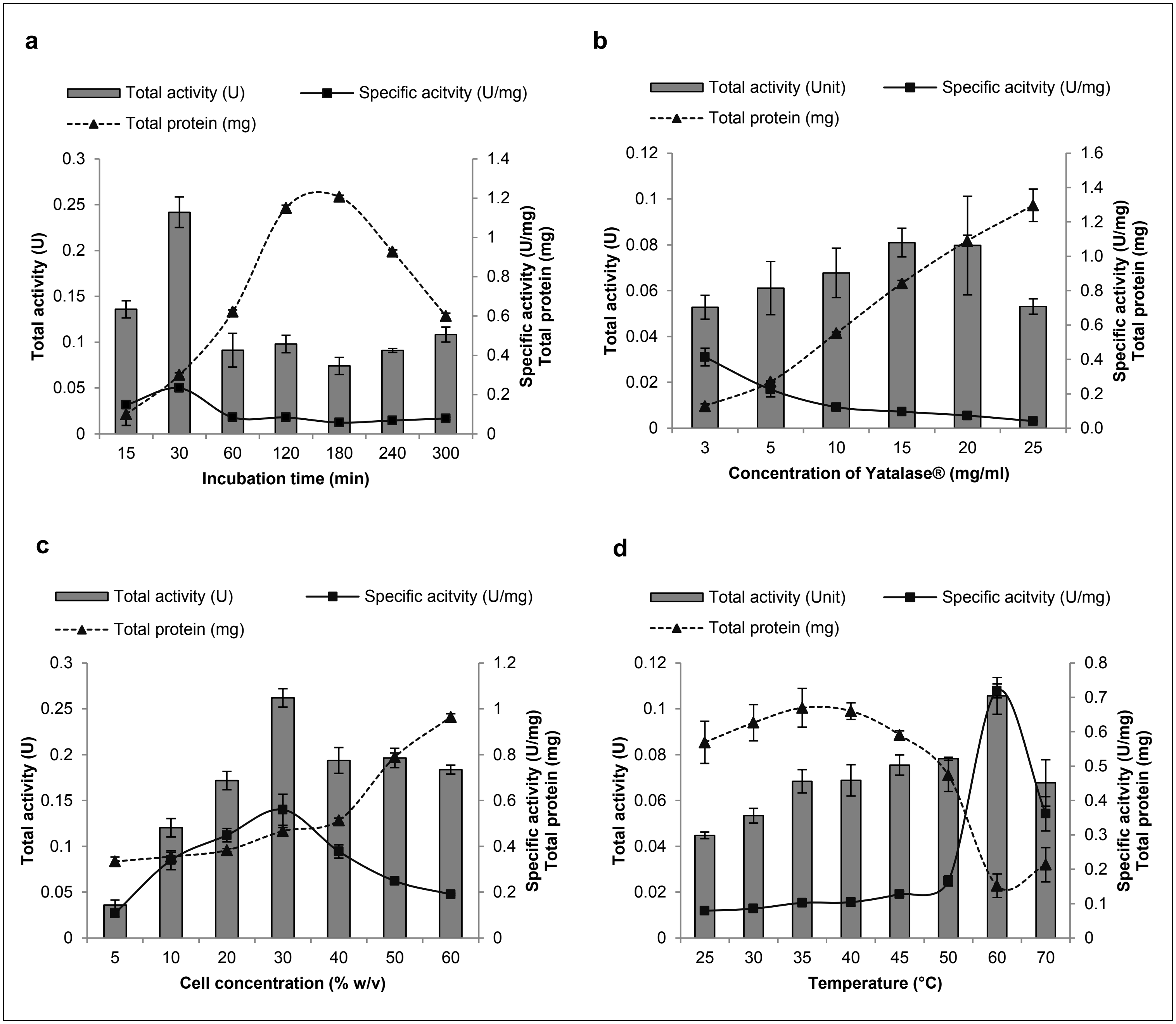

Additional file 1

Optimization of the condition of enzymatic lysis on the recovery of GAD by using Yatalase®. (a) Effect of incubation time (min) of Yatalase® (10 mg/ml) on 10% (w/v) of mycelia at 30 ºC in 50 mM Citrate buffer, pH 5.5. (b) Effect of concentration of lytic enzyme on mycelia (10% w/v) at 30 ºC for 60 min. (c) Effect of cell concentration (% w/v) on the enzymatic lysis of Yatalase® (10 mg/ml) at 30 ºC for 60 min. (d) Effect of incubation temperature on the enzymatic lysis of Yatalase® (10 mg/ml) at 10% (w/v) of mycelia for 60 min. Results represent the mean (± SD) of three experiments.
